# An Ultrasensitive Label-Free Aptasensor for Insulin Detection Assisted by Exonuclease III and 2-Aminopurine

**DOI:** 10.3390/molecules31122173

**Published:** 2026-06-21

**Authors:** Dongdong Shi, Yanhua He, Guiqin Yan

**Affiliations:** 1 School of Chemistry and Chemical Engineering, Shanxi Normal University, Taiyuan 030031, China; 118111005@stu.sxnu.edu.cn; 2 School of Food Science, Shanxi Normal University, Taiyuan 030031, China; 3 School of Life Sciences, Shanxi Normal University, Taiyuan 030031, China

**Keywords:** 2AP, exonuclease III, aptasensor, label-free, fluorescence, insulin

## Abstract

We designed a label-free fluorescent aptasensor assisted by exonuclease III (Exo III) for sensitive insulin (Ins) detection. The method has high sensitivity, anti-interference properties and repeatability. Additionally, the label-free fluorescent aptasensor assisted by Exo III used to detect Ins has not been reported on yet. In this study, we connected a modified DNA sequence to the 5′ end of an aptamer, modifying it into a hairpin structure and exposing 11 nucleotides at the 3′ end containing the base adenine (A). The A was substituted with base 2-aminopurine (2AP) to provide a label-free stable hairpin fluorescent probe (2AP-hairpin probe). This strategy took advantage of the high binding affinity of the Ins aptamer and the susceptibility of 2AP to the local base stacking environment. When Ins is added to the detection system, the 2AP-hairpin probe binds to Ins, adopts a folded state, and blocks Exo III’s access to the binding site for cutting DNA. 2AP cannot be released, and the fluorescence of the 2AP-hairpin probe/cDNA/Ins/Exo III system cannot be restored. Ins detection is achieved by comparing changes in the fluorescent intensity before and after adding Ins to the detection system. The detection limit of the aptasensor is as low as 1.62 nM with a linear range of 3–130 nM. Furthermore, it is able to selectively and directly detect Ins in biological fluids, demonstrating significant clinical application value and research significance.

## 1. Introduction

Ins is one of the most important peptide hormones in mammals [1]; it maintains normal blood sugar levels by regulating the metabolism of carbohydrates, proteins, lipids, and other organic substances and promotes cell division and growth through a mitogenic effect [2]. Ins is the most crucial indicator when evaluating β-cell endocrine function [3], and abnormal Ins secretion is closely related to obesity, diabetes, hypoglycemia, insulinoma, insulin resistance syndrome, and other diseases [4]. Long-term abnormal insulin secretion may cause neurodegenerative diseases, myocardial infarction, and even cancer [1]. Therefore, it is very important to develop a sensitive and efficient method for Ins detection.

Various immunoassay [5,6], electrochemical [7,8,9], photochemical [10,11,12], and chromatographic [13,14] Ins detection methods have been developed. However, each technique has its drawbacks. In the immunoassay method, the enzyme easily cross-reacts with proinsulin and C-peptide, and the assays cannot meet the demands of field testing. Electrochemical methods can provide a portable solution for insulin detection, but electrochemical sensors are easily hindered by the kinetics involved in the slow oxidation of interfering molecules, such as glucose, uric acid, and ascorbic acid in biological fluids, and they have limited stability and sensitivity [15]. The chromatographic method involves complicated detection schemes and pretreatment steps; is time-consuming, laborious, and expensive; and has limited sensitivity. Thus, it has difficulty detecting low insulin concentrations in body fluids [5,15]. Fluorescent biosensors based on nucleic acid aptamers can solve the above problems, as they have the advantages of high stability and sensitivity and are quick, convenient, and simple to operate [4,11,16,17]. However, fluorescent probe labeling reduces the affinity of the aptamer to the target and increases the complexity of the test [16,17,18,19].

By using the base 2AP as the fluorescent probe for the aptamer sequence, we can circumvent the labeling process and avoid the decrease in aptamer affinity caused by the modification [20]. The base A in the oligonucleotide can be replaced with 2AP, which is able to perform base complementary pairing with thymine (T) [21]. Moreover, 2AP can produce a strong fluorescent signal, the intensity of which is highly dependent on the state of the DNA strand [18,21]. The fluorescent intensity is strongest when 2AP is in a free state and less strong when it is inserted into single-stranded DNA (sDNA); however, when 2AP is within double-stranded DNA (dsDNA), the fluorescence is significantly quenched to minimal levels as a result of complementary base pairing [22]. Exo III can degrade the blunt and concave ends of the 3′ end of dsDNA but has almost no sDNA-degrading activity [23,24]. Moreover, Exo III can more potently degrade hairpin-structure DNA with a greater stem length [25]. Taking these factors into consideration, our objective in this study was to couple a modified DNA (mDNA) sequence to the 5′ end of a nucleic acid aptamer sequence to form a stable hairpin structure with 5-base pairing. The sequence containing A at the 3′ end can be exposed to protect it from degradation by Exo III, and the exposed A can be replaced with 2AP to form a fluorescent probe (2AP-hairpin probe). The ability of Exo III to variably degrade DNA strands in different states can be used to prepare a label-free fluorescence aptasensor [26]. Using this method, we aimed to develop an aptasensor to detect Ins in biological fluids, with potential applications in clinical diagnosis and related basic research.

## 2. Results and Discussion

### 2.1. Construction of Fluorescent Aptamer Probe for Insulin

In this study, a systematic classification and performance comparison were conducted for 2-AP [27], 2-Aza-2AP (2-Aza-2-amino-2′-deoxyribonucleoside) [28], 8VA (8-Vinyladenine) [29], DAP (2,6-Diaminopurine) [30], 6-MI (6-Methylisoxanthopterin) [31], 2PyG(2-Pyridylguanine) [32], and 4CIN (4 Cyanoindole 2′ deoxyribonucleoside) [33] based on reported classic fluorescent nucleoside analogs. Adenine-based fluorescent nucleosides showed excellent overall performance, but each had inherent defects: 2-Aza-2AP had a high synthesis cost, 8VA was prone to severe fluorescence quenching, and DAP exhibited significant background interference under ultraviolet excitation. Guanine-based fluorescent nucleosides (6-MI, 2PyG) were only suitable for guanine site modification and easily disrupted the aptamer conformation, while 4CIN lacked hydrogen bond matching with natural bases. Among them, 2-AP had a molecular structure highly consistent with natural adenine, causing the least perturbation to the aptamer and optimal hydrogen bond matching, making it an ideal candidate for the fluorescent labeling of insulin aptamers.

**Figure 1:**
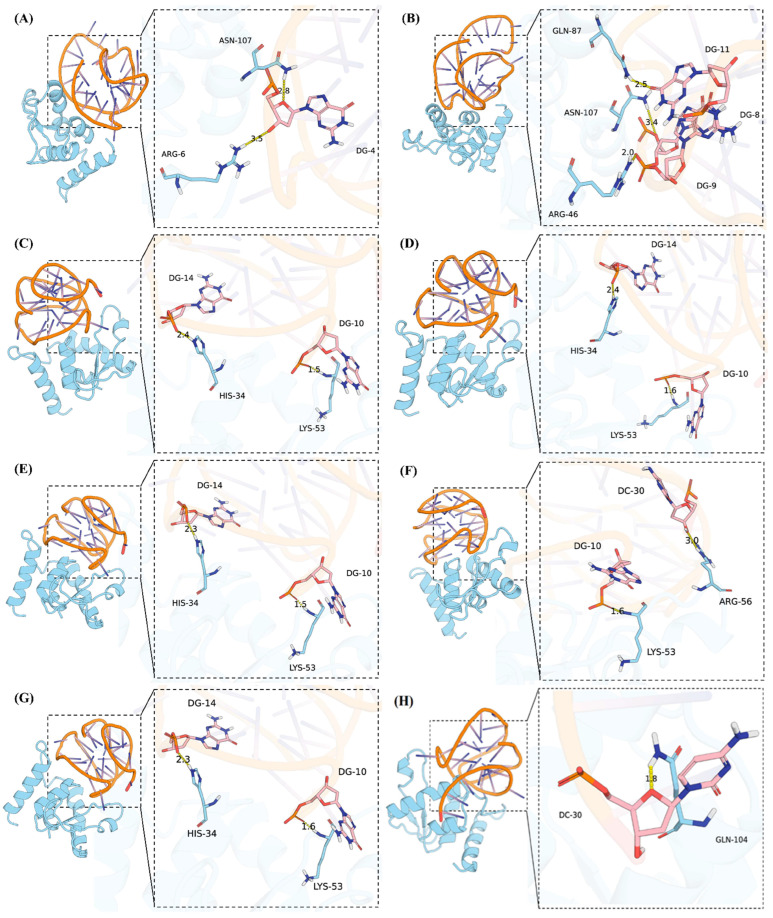
(**A**) Docking pattern of unmodified 2AP aptamer with insulin; (**B**) 2AP-modified aptamer docked with insulin; (**C**) 2-aza-2-AP-modified aptamer docked with insulin (**D**) 8-VA-modified aptamer docked with insulin; (**E**) 6-MI-modified aptamer docked with insulin; (**F**) 2PyG-modified aptamer docked with insulin; (**G**) 4CIN-modified aptamer docked with insulin; (**H**) DAP-modified aptamer docked with insulin (blue represents insulin, orange represents aptamer, and yellow dashed lines indicate hydrogen bond interactions.).

To further screen the optimal fluorescent probe suitable for insulin aptamers, molecular docking simulations were performed using HDOCKlite v1.1 [34], and PyMOL2.5.3 [35] was used to visually analyze the optimal binding conformation so as to clarify the molecular interaction mechanism between insulin and fluorescently modified aptamers. As shown in Figure 1 and Table 1, the native insulin aptamer exhibited the strongest binding stability with a binding energy of −326.99 kcal/mol when bound to insulin. Among all modified probes (Figure 1A), the 2-AP-modified hairpin probe had a binding energy of −321.38 kcal/mol, which was the closest to the native aptamer in terms of binding potential. This probe could form a multi-site synergistic hydrogen bond network with insulin, including Arg46-dG9 (2.0 Å), Gln87-dG11 (2.5 Å), and Asn107-dG8 (3.4 Å) (Figure 1B). The above multiple hydrogen bonds formed a synergistic stabilization effect in space, significantly enhancing the binding strength at the insulin–aptamer interface and effectively maintaining the local and overall conformational stability of the complex. In contrast, the adenine-based modified probes 2-aza-2AP (−316.11 kcal/mol) (Figure 1C) and 8VA (−312.43 kcal/mol) (Figure 1D) showed a less negative binding energy compared with the native aptamer, and the integrity of the hydrogen bond network at the binding interface was decreased. However, the docking scores of the 6-MI (−301.81 kcal/mol) (Figure 1E), 2PyG (−293.26 kcal/mol) (Figure 1F), 4CIN (−285.46 kcal/mol) (Figure 1G), and DAP (−284.67 kcal/mol) (Figure 1H) were less negative. Mechanistic analysis indicated that such modifications would destroy the key hydrogen bond sites of the aptamer, weaken the polar interaction between K53 and the DNA phosphate backbone, and introduce additional steric hindrance, ultimately leading to a significant reduction in the target recognition ability of the probe. In conclusion, 2-AP modification barely alters the spatial conformation and target binding capacity of the insulin aptamer, making it the optimal choice for preparing fluorescent aptamer probes against insulin.

Increasing the amount of base pairing in the long-chain oligonucleotide sequence reduces the reaction rate [36]. To shorten the oligonucleotide base-pairing time, we modified the aptamer into a hairpin structure, as shown in Figure S1. A 5-base modified sequence (5′-ACCAA-3′) was connected to the 5′ end of the aptamer, and the sequence containing A at the 3′ end was exposed. As an adenine A, 2AP can replace adenine without damaging the DNA structure and is linked to flanking bases (guanine, thymine) by phosphodiester bonds to form a 2AP fluorescent probe with a stable hairpin structure (2AP hairpin probe).

### 2.2. Working Principle of This Aptasensor

**Scheme 1:**
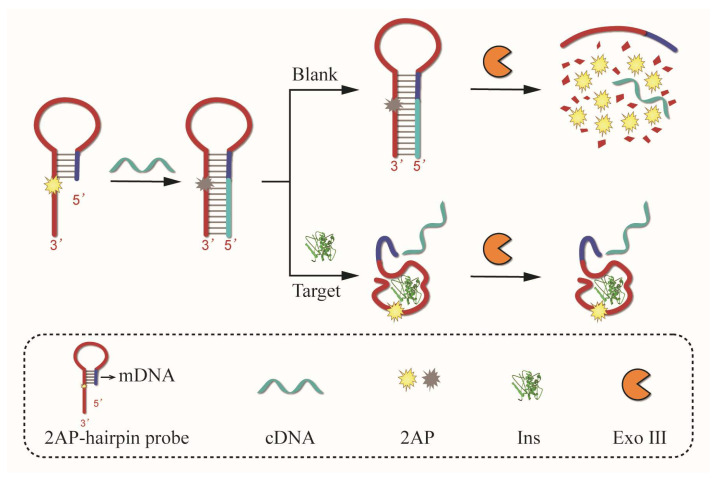
Mechanism of sensing related to the proposed label-free aptasensor for Ins detection.

The principle of detection is illustrated in Scheme 1. This study establishes a signal-loss-based fluorescence assay for insulin detection. The 2AP-hairpin probe combines with the cDNA fragment of the 3′-end protruding sequence to form a hairpin structure with blunt ends and a longer stem (2AP-hairpin probe/cDNA), and the 2AP-hairpin probe fluorescence is significantly quenched. After Exo III is added to the detection system, it degrades the stem of the hairpin structure from the 3′ end; 2AP is restored to a high level. The aptamer has a more potent affinity to the target compared with its complementary sequence; thus, when Ins is added to the detection system, the 2AP-hairpin probe binds to Ins through hydrogen bonding [12] and adopts a folded state. This structure masks the recognition and cleavage sites of Exo III, thereby inhibiting enzymatic hydrolysis of the probe and restricting the liberation of free 2-AP. As a result, the fluorescence signal cannot be restored and stays at a low steady state. Accordingly, the quantitative detection of insulin is accomplished based on the concentration-dependent attenuation of fluorescence.

**Figure 2:**
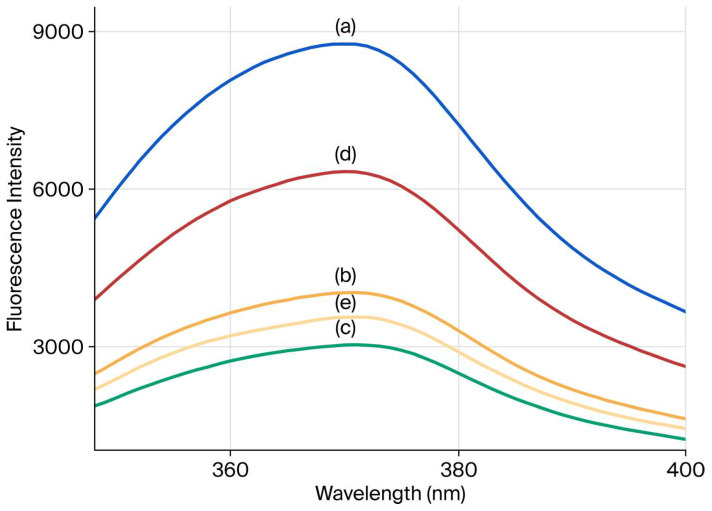
Fluorescent spectra of 2AP (**a**); 2AP-hairpin probe (**b**); 2AP-hairpin probe/cDNA (**c**); 2AP-hairpin probe/cDNA/ Exo III (**d**); 2AP-hairpin probe/cDNA/Ins/Exo III (**e**). Concentrations of 2AP, 2AP-hairpin probe, cDNA, Ins, and Exo III were 20 nM, 20 nM, 20 nM, 60 nM, and 0.2 U/uL, respectively.

In order to test the feasibility of this approach, the fluorescence intensity under different conditions was measured. As presented in Figure 2, the free 2AP in the solution emitted strong fluorescence (Figure 2a), and after inserting 2AP into the aptamer hairpin sequence, the fluorescence of the 2AP-hairpin probe was weakened due to photoinduced electron transfer caused by the base stacking effect (Figure 2b) [37,38]. The cDNA sequence of the 3′ end of the aptamer was added to the detection system; 2AP bound to base T, and the fluorescence of the 2AP-hairpin probe/cDNA system was considerably quenched (Figure 2c) [39,40] at a rate of 65%. The results reveal that base stacking combined with hydrogen bonding reduces the oscillator strength for the fluorescence transition, hence the fluorescence quantum yield [41]. Then, Exo III was added to the detection system, and it degraded the dsDNA and dissociated single nucleotides sequentially in the 3′→5′ direction [42,43]. The 2AP inserted into the 2AP-hairpin probe was released, and the fluorescence of the 2AP-hairpin probe/cDNA/Exo III system was remarkably restored (Figure 2d) with a recovery rate of 72%. However, by adding Ins to the detection system and then adding Exo III, the aptamer sequence of the 2AP-hairpin probe specifically bound to Ins and adopted a folded state, Exo III lost the binding site for degrading dsDNA, and the fluorescence intensity in the detection system could not continue to increase (Figure 2e) [44]. The more Ins was added, the weaker the fluorescence intensity of the 2AP-hairpin probe/cDNA/Ins/Exo III system became compared with that of the 2AP-hairpin probe/cDNA/Exo III system, facilitating Ins detection.

### 2.3. Optimization of the Experimental Circumstances

**Figure 3:**
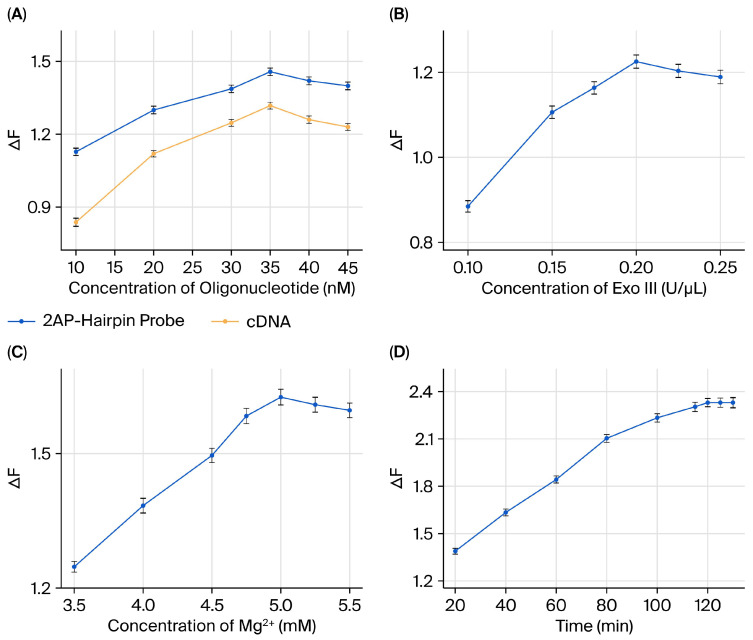
Influence of (**A**) concentration of 2AP-hairpin probe and concentration of cDNA; (**B**) concentration of Exo III; (**C**) concentration of Mg^2+^; (**D**) time course of Exo III degradation of dsDNA.

The fluorescence intensity of 2AP is strongly dependent on the environment of the inserted nucleotide sequence [40,45]. We optimized the concentrations of the 2AP-hairpin probe and complementary cDNA in the detection system. As illustrated in Figure 3A, the ΔF value tended to increase with increasing 2AP-hairpin probe concentration until it reached 35 nM, at which point the 2AP-hairpin probe was added to the detection system, excessive base stacking affected the fluorescent intensity of 2AP [46], and the ΔF value no longer increased. We used the same method to optimize the cDNA concentration and found that when the same concentration of cDNA was selected, it combined with the 2AP-hairpin probe to form dsDNA with the highest efficiency and the best response. Therefore, we chose 35 nM as the optimal concentration for both the 2AP-hairpin probe and cDNA. The rate at which Exo III degraded dsDNA was strongly dependent on the Exo III-to-dsDNA concentration ratio and the concentration of Mg^2+^ [47]. As shown in Figure 3B,C, when Exo III was at 0.2 U/µL, all dsDNA generated in the coupling stage was degraded, and the optimal concentration of Mg^2+^ was 5 mM. On this basis, we optimized the response times required for each stage. As shown in Figure S2, the best reaction times for the 2AP-hairpin probe to cDNA coupling (time 1) and aptamer binding of Ins (time 2) were both 20 min. In the Exo III degradation of the 2AP-hairpin probe/cDNA, only a few nucleotides were removed each time Exo III binded to the substrate [47]; therefore, as the reaction time increased, the degradation rate increased, and Exo III fully degraded dsDNA in 120 min (Figure 3D). Hence, in the follow-up experiments, we chose 120 min as the optimal degradation time.

### 2.4. Aptasensor Sensitivity Detection

**Figure 4:**
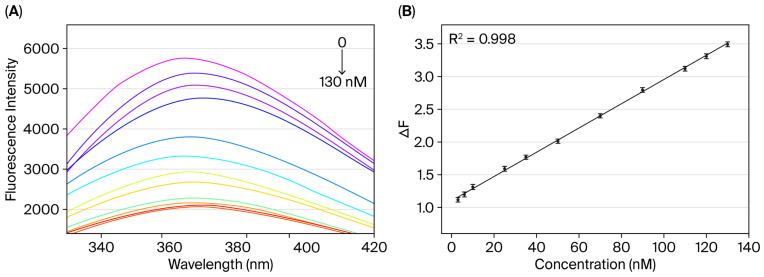
(**A**) Fluorescence emission spectra of the Exo III-assisted aptasensor at different concentrations of Ins (0, 3, 6, 10, 25, 35, 50, 70, 90, 110, 120, and 130 nM); (**B**) calibration plot of the aptasensor with insulin concentrations ranging from 0 to 130 nM.

Under optimized experimental circumstances, the modification in the spectrum of fluorescence emission at 370 nm was observed after adding different Ins concentrations (0–130 nM) to the detection system. From Figure 4A, it is clear that the fluorescence intensity related to the detection system gradually reduced with increasing Ins concentration. Moreover, the association was linear in the 3–130 nM detection range, with a linear equation of ΔF = 0. 0131C[Ins] + 1.0605 (R^2^ = 0.998) (Figure 4B). The detection limit (LOD) was 1.62 nM, which was calculated using the equation LOD = 3σ/s, where σ represents the standard deviation of 12 parallel assessments without Ins in the detection system and s indicates the slope of the calibration curve.

### 2.5. Aptasensor Specificity, Anti-Interference, and Reproducibility Detection

**Figure 5:**
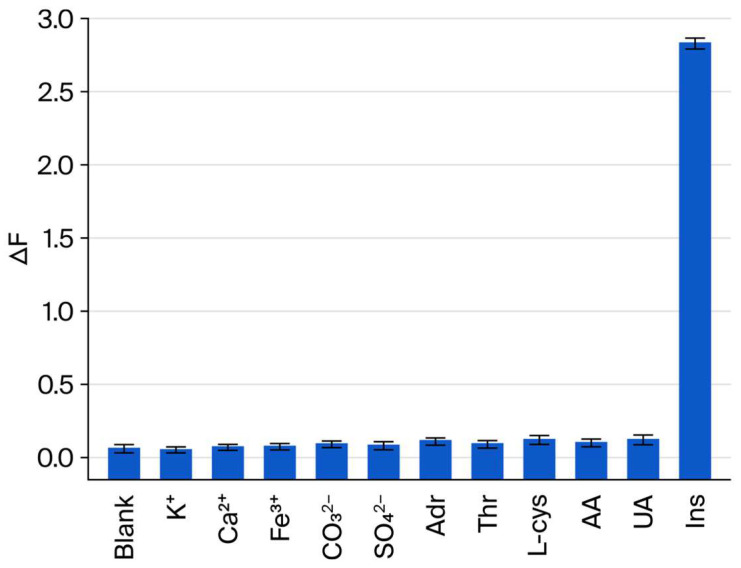
The specificity of the proposed probe to Ins and other interfering substances.

To determine the specificity and anti-interference of the sensor, we compared the relative fluorescent intensity changes in the detection system after adding 50 nM lns and 500 nM nonspecific substances that may exist in biological fluids: Adr, Thr, L-cys, AA, and UA, as well as interfering ions (K^+^, Ca^2+^, Fe^3+^, CO_3_^2−^, and SO_4_^2−^). As illustrated in Figure 5, the value of ΔF significantly increased after lns was added to the detection system. However, nonspecific substances and interfering substances, even when their concentration is 10 times higher than that of insulin; the change in ΔF was almost the same as that of the blank control group (<5%). All of the observations above clearly indicated the high specificity and anti-interference of the proposed probe for Ins detection.

We also tested the repeatability of the sensor by measuring the intensity of the fluorescent response to the addition of 60 nM Ins to the detection system five consecutive times, and we calculated the relative standard deviation (RSD) to be approximately 3.56%. This result is similar to other lns detection aptasensors, indicating that the repeatability of the biosensor is within the acceptable range.

### 2.6. Detection of Ins in Biological Fluids

We further studied the performance of the aptasensor in the detection of Ins in biological fluids. Different concentrations (5, 30, and 100 nM) of Ins were added to fetal bovine serum, which was then diluted 10 times with Tris-HCl (pH 7.4). Modifications in the fluorescence intensity were observed, and the spike recovery rate was calculated, as shown in Table 2. The spike recovery rates ranged from 96.3% to 98.4%, indicating that the designed aptasensor has good practicability and can effectively detect Ins in biological fluids.

### 2.7. Superiority of the Aptasensor

The performance of this aptasensor was compared with other aptamer-based methods with regard to the detection of Ins (Table 3). The method developed was found to have higher sensitivity than the other fluorescence-based aptasensors [12,48]. In addition, our technique had a broader linear range than the two techniques [9,49], and there is no need to prepare complex metal nanomaterials, such as MB-IBA [7], NiO/Fe_2_O_3_ [8], CdSe/ZnS QDs [12], AuNPs [13] in the detection process. Furthermore, base A can be easily replaced with 2AP without the use of complicated coupling labeling methods. With a total assay time of 2.67 h, the present strategy employs a probe featuring facile preparation and no additional modification. Its detection efficiency surpasses most reported techniques and is comparable to commercially available ELISA kits (Table S1). Therefore, the aptasensor assisted by Exo III developed in this study could facilitate highly sensitive, environmentally friendly, and efficient detection of Ins in biological fluids.

### 2.8. Analysis of Detection Range Adaptability and Application Prospects

Relying on the favorable comprehensive performance of the aptasensor, this system exhibits a linear range of 3–130 nM and a limit of detection of 1.62 nM. The fasting physiological insulin level in healthy subjects is 20–150 pM, and the postprandial insulin peak can reach 5–10 times the fasting level. This concentration range is below the lower limit of quantification of our method, so the system is not suitable for accurately quantifying basal fasting and postprandial insulin in healthy populations. In contrast, our method mainly focuses on pathological hyperinsulinemia, particularly in two typical clinical scenarios: insulin autoimmune syndrome [53,54,55], and exogenous insulin abuse [56]. The linear range is highly compatible with these samples, enabling sensitive and accurate quantification without the need for sample dilution, thus providing strong support for clinical screening and auxiliary diagnosis of these special endocrine disorders.

At the same time, this method has a wide range of applications in basic medical research. For stem cell-derived insulin-producing cell models, this method can quantitatively analyze insulin secretion behavior under glucose stimulation, objectively evaluate cell differentiation maturity and physiological function, and support research on diabetes cell therapy [57,58]. In the development of novel hypoglycemic drugs, the wide linear range can fully cover insulin concentration changes in pharmacodynamic experiments, effectively analyze the regulatory effects of drugs on pancreatic β-cell function, and support drug screening and mechanism analysis [59].

## 3. Materials and Methods

### 3.1. Materials and Chemicals

Fetal bovine serum, adrenaline (Adr), thrombin (Thrombin, Thr), L-cystine (L-cys), uric acid (UA), and ascorbic acid (AA) were purchased from Yuan Ye Bio-Technology Co., Ltd. (Shanghai, China); Tris reagent and 2AP base were obtained from Sigma-Aldrich (St. Louis, MO, USA). The sequence of the 2AP modified aptamer (5′-GGT GGT GGG GGG GGT TGG TAG GGT GTC TTC-3′) [44] and all other oligonucleotide sequences related to the synthetic hairpin structure (Table 4) were prepared and purified using HPLC by Sangon Biotech (Shanghai, China), protected through 1 × Tris-EDTA buffer (10 mM Tris, 1 mM EDTA) and stored at −20 °C until required. Exo III (200 U/µL) was acquired from Sangon Biotech (Shanghai) and kept in the freezer at −20 °C until further use. HCl, MgCl_2_, and other reagents employed in the current investigation were of analytical grade and were bought from Guangfu Chemicals Company (Tianjin, China). Ultrapure water was utilized to configure all experimental reagents (18.2 MΩ/cm, Milli-Q Plus system, Millipore, Burlington, MA, USA).

### 3.2. Instrumentation

Fluorescence spectra were collected using an F98 Fluorescence spectrophotometer (Lengguang Technology Co., Ltd., Shanghai, China). The fluorescence spectra were recorded from 320 nm to 450 nm at an excitation wavelength of 310 nm. Slit widths for excitation and emission were 5 nm and 10 nm, respectively. All fluorescence detection experiments were carried out at ambient temperature.

### 3.3. Insulin Detection

The specific detection process was as follows: 35 nM of the 2AP-hairpin probe and an equal concentration of cDNA were added to the colorimetric tube, which was shaken thoroughly, and the mixture reacted for 20 min at 37 °C to allow a hairpin structure with blunt ends and a longer stem to form (2AP-hairpin probe/cDNA), and 2AP fluorescence was significantly quenched. We then added a series of Ins concentrations (0, 3, 6, 10, 25, 35, 50, 70, 90, 110, 120, and 130 nM) to the reaction system, which was kept at room temperature for 20 min to allow the aptamer to fully bind to the target. We subsequently added 0.2 U/µL Exo III and 5 mM Mg^2+^ to the detection system, which was then kept in a water bath at 37 °C for 120 min to allow Exo III to degrade the quenched hairpin nucleic acid sequence, which does not bind to the target. All stages of the insulin detection experiment were performed in a 10 mL colorimetric tube, and the volume of the reaction solution was maintained at 5 mL with Tris-HCl (pH 7.4) during the reaction (Detailed procedures are provided in Supplementary Materials). To reduce background interference, the output value of fluorescence was demonstrated as the relative fluorescence intensity (ΔF) where ΔF = (F_0_ − F_1_)/(F − F_1_), and F and F_0_ represent the fluorescence intensity with and without the addition of Ins to the system of detection, respectively. F_1_ refers to the fluorescence intensity of the system containing all components except Exo III. The fluorescence intensity of the detection system was assessed 3 times (ΔF > 1, with a direct positive correlation to insulin concentration).

## 4. Conclusions

We designed a label-free fluorescent aptasensor assisted by Exo III and successfully applied it to the sensitive detection of Ins. This system can facilitate the screening of clinical disorders associated with hyperinsulinemia and is also applicable to research including islet cell analysis and drug screening. The experimental results demonstrate that this aptasensor has many advantages for Ins detection in biological fluids. For example, the method has excellent selectivity, anti-interference properties, repeatability, and practicability. Although the detection limit of our aptasensor is not as low as some other Ins detection methods designed in recent years, the detection process does not require complex sample pretreatment, the preparation of complex nanomaterials, or coupling reagents, and no secondary pollutants are introduced. Future studies will further explore the application value of this sensor in insulin detection for healthy populations, aiming to provide technical support for clinical diagnosis and basic biomedical research. Meanwhile, this detection strategy can also be extended to analyze other target substances.

## Figures and Tables

**Table 1:** Affinity analysis results between insulin and aptamers.

Target	Ligand	Docking_Score (kcal/mol)
insulin	Aptamer	−326.99
insulin	2AP-hairpin probe	−321.38
insulin	2-aza-2AP	−316.11
insulin	8VA	−312.43
insulin	6-MI	−301.81
insulin	2PyG	−293.26
insulin	4CIN	−285.46
insulin	DAP	−284.67

(A more negative binding affinity value indicates a stronger binding possibility).

**Table 2:** Ins determination in fetal bovine serum samples by the fabricated aptasensor.

Bovine Serum Samples Number	Added Concentration (nM)	Detected Concentration (nM)	Recovery (%) (Mean ± s; n = 3)
1	5	4.92	98.4 ± 2.7
2	30	29.2	97.3 ± 3.8
3	100	96.3	96.3 ± 4.2

**Table 3:** Comparison of the Ins detection performance of aptamers developed in previous years.

Method	Probe	Linear Range (nM)	Detection Limit (nM)	Time (h)	Reference
Electrochemical	MB-IBA	0.01–10	1.0 × 10^−4^	54	[7]
Electrochemical	NiO/Fe_2_O_3_	1.72 × 10^−7^–1.72	1.6 × 10^−7^	1.5	[8]
Electrochemical	AuNP–DAb	0–5	1.2 × 10^−2^	4.08	[9]
Electrochemical	POCT	1.72 × 10^−2^–1.722 × 10^−1^	1.72 × 10^−2^	15	[50]
Fluorescence	Fc-IGA3@COF/β-CD@AuNCs	1.75 × 10^−6^–175	1.6 × 10^−6^	2	[11]
Fluorescence	CdSe/ZnS QDs	4.3–206.4	2.74	4.33	[12]
Fluorescence	Transduction	0–100	9.97	15	[48]
Fluorescence	R-CDs@apt/GO	1.3–150	1.1	24	[49]
Chemiluminescence	ssDNA-AuNPs/ IGA3/SiO_2_@GOAG	7.5 × 10^−3^–5	1.6 × 10^−3^	24	[13]
ELISA	Thio-NAD	0–0.048	2.28 × 10^−3^	12	[51]
ELISA	C-MNP-IA	0.01	0.01–10	2	[52]
Fluorescence	2AP	3–130	1.62	2.67	This work

**Table 4:** Sequences of the used oligonucleotides.

Type	Section	Sequence(5′-3′)
2AP-hairpin probe	Aptamer	GGT GGT GGG GGG GGT TGG TA**G GGT GTC TTC**
	mDNA	ACCAA
Complementary strand	cDNA	GAAGACACCCT

The underlined base A indicates substitution by 2AP. The bold region of aptamer shows the exposed sequence of the hairpin structure.

## Data Availability

The original contributions presented in this study are included in the article. The raw data supporting the conclusions of this article will be made available by the authors on request. Inquiries can be directed to the corresponding author.

## Supplementary Materials

The following supporting information can be downloaded at: https://www.mdpi.com/article/10.3390/molecules31122173/s1, S1. Insulin detection; Figure S1 Secondary structure of 2AP-hairpin probe; Figure S2 The best reaction times for the 2AP-hairpin probe to cDNA coupling (time 1) and aptamer binding of Ins (time 2); Table S1 Comparison with commercial colorimetric enzyme-linked immunosorbent assay (ELISA) kits for insulin detection.

## Abbreviations

The following abbreviations are used in this manuscript:

Ins	Insulin
Exo III	Exonuclease III
2AP	2-Aminopurine
A	Adenine
2AP-hairpin probe	The exposed A replaced with 2AP to form a fluorescent probe
T	Thymine
sDNA	Single-Stranded DNA
dsDNA	Double-Stranded DNA
mDNA	Modified DNA
Adr	Adrenaline
Thr	Thrombin
L-cys	L-cystine
UA	Uric acid
AA	Ascorbic acid
ΔF	The relative fluorescence intensity
2-Aza-2AP	2-Aza-2-amino-2′-deoxyribonucleoside
8VA	8-Vinyladenine
DAP	2,6-Diaminopurine
6-MI	6-Methylisoxanthopterin
2PyG	2-Pyridylguanine
4CIN	4 Cyanoindole-2′-deoxyribonucleoside
cDNA	The complementary chain

## References

[1] Xu M., Luo X., Davis J.J. (2013). The label free picomolar detection of insulin in blood serum. Biosens. Bioelectron..

[2] Taib M., Tan L.L., Abd Karim N.H., Ta G.C., Heng L.Y., Khalid B. (2020). Reflectance aptasensor based on metal salphen label for rapid and facile determination of insulin. Talanta.

[3] Singh V., Nerimetla R., Yang M., Krishnan S. (2017). Magnetite-Quantum Dot Immunoarray for Plasmon-Coupled-Fluorescence Imaging of Blood Insulin and Glycated Hemoglobin. ACS Sens..

[4] Bisker G., Bakh N.A., Lee M.A., Ahn J., Park M., O’Connell E.B., Iverson N.M., Strano M.S. (2018). Insulin Detection Using a Corona Phase Molecular Recognition Site on Single-Walled Carbon Nanotubes. ACS Sens..

[5] Xu Z., He H., Nie R. (2025). A cucurbit [7] uril-mediated crosslinking-amplified microchannel resistance immunoassay via magnetic interception for point of care testing of insulin. Talanta.

[6] Henry H., Lannoy D., Simon N., Seguy D., D’Herbomez M., Barthelemy C., Decaudin B., Dine T., Odou P. (2017). Immunoassay quantification of human insulin added to ternary parenteral nutrition containers: Comparison of two methods. Anal. Bioanal. Chem..

[7] Zhao Y., Xu Y., Zhang M., Xiang J., Deng C., Wu H. (2019). An electrochemical dual-signaling aptasensor for the ultrasensitive detection of insulin. Anal. Biochem..

[8] Gu C., Liu Y., Hu B., Liu Y., Zhou N., Xia L., Zhang Z. (2020). Multicomponent nanohybrids of nickel/ferric oxides and nickel cobaltate spinel derived from the MOF-on-MOF nanostructure as efficient scaffolds for sensitively determining insulin. Anal. Chim. Acta.

[9] Nandhakumar P., Munoz San Martin C., Arevalo B., Ding S., Lunker M., Vargas E., Djassemi O., Campuzano S., Wang J. (2023). Redox Cycling Amplified Electrochemical Lateral-Flow Immunoassay: Toward Decentralized Sensitive Insulin Detection. ACS Sens..

[10] Liu C., Han J., Zhang J., Du J. (2019). Novel detection platform for insulin based on dual-cycle signal amplification by Exonuclease III. Talanta.

[11] Chen W., Leng H., Li J., Zhang H., Zhang Y., Chen X. (2026). Label-free aptasensor for ultrasensitive detection of insulin via AuNCs induced fluorescence “on-off” of covalent organic framework. Sens. Actuators B Chem..

[12] Yang J., Zhang Z., Yan G. (2018). An aptamer-mediated CdSe/ZnS QDs@graphene oxid composite fluorescent probe for specific detection of insulin. Sens. Actuators B Chem..

[13] Sun Y., Lin Y., Sun W., Han R., Luo C., Wang X., Wei Q. (2019). A highly selective and sensitive detection of insulin with chemiluminescence biosensor based on aptamer and oligonucleotide-AuNPs functionalized nanosilica @ graphene oxide aerogel. Anal. Chim. Acta.

[14] Reverter-Branchat G., Groessl M., Nakas C.T., Prost J.C., Antwi K., Niederkofler E.E., Bally L. (2020). Rapid quantification of insulin degludec by immunopurification combined with liquid chromatography high-resolution mass spectrometry. Anal. Bioanal. Chem..

[15] Chong J.Q., Md Shakhih M.F., Vijayam B., Marimuthu C., Ching C.T.S., Abdul Wahab A. (2025). Insulin Quantification Through Electrochemical and Optical Aptasensors: A Review. Ann. Biomed. Eng..

[16] Zhao X., Dai X., Zhao S., Cui X., Gong T., Song Z., Meng H., Zhang X., Yu B. (2021). Aptamer-based fluorescent sensors for the detection of cancer biomarkers. Spectrochim. Acta Part A Mol. Biomol. Spectrosc..

[17] Li Z., Yang Y., Tang Q., Zeng Y., Wang H., Li L. (2025). Ultrasensitive determination of insulin by an unlabelled fluorescent aptasensor based on Fe(3)O(4)@SiO(2) and an oxadiazole compound with AIEE activity. Mikrochim. Acta.

[18] Liu X., Dong L., Wang L., Xu H., Gao S., Zhong L., Zhang S., Jiang T. (2019). 2-Aminopurine modified DNA probe for rapid and sensitive detection of l-cysteine. Talanta.

[19] He Y., Yu Y., Wen X., Shi Y., Wu J., Guan Z., Cui M., Xiao C. (2020). A quencher-free 2-aminopurine modified hairpin aptasensor for ultrasensitive detection of Ochratoxin A. Spectrochim. Acta A Mol. Biomol. Spectrosc..

[20] Yang F., Li S., Wu J., Liu S. (2024). 2-Aminopurine-based quencher-free DNA tweezers with fluorescence properties well tuned by surrounding bases. Anal. Methods.

[21] Hogan M.E., LeGrice S.F., Wemmer D.E. (1987). Base pairing and mutagenesis: Observation of a protonated base pair between 2-aminopurine and cytosine in an oligonucleotide by proton NMR. Proc. Natl. Acad. Sci. USA.

[22] Liao R., He K., Chen C., Chen X., Cai C. (2016). Double-Strand Displacement Biosensor and Quencher-Free Fluorescence Strategy for Rapid Detection of MicroRNA. Anal. Chem..

[23] Wang D., Yan S.L., Yang X.T., Yang C.G., Xu Z.R. (2025). Highly sensitive detection of miRNA based on a quencher-free fluorescence strategy with dual-signal amplification. Anal. Bioanal. Chem..

[24] Liu W.J., Xu Q., Ma F., Li C.C., Zhang C.Y. (2018). Exonuclease III-assisted multiple cycle amplification for the sensitive detection of DNA with zero background signal. Analyst.

[25] Jiang X., Liu H., Khusbu F.Y., Ma C., Ping A., Zhang Q., Wu K., Chen M. (2018). Label-free detection of exonuclease III activity and its inhibition based on DNA hairpin probe. Anal. Biochem..

[26] Zhao Q., Zhang G., Lu D., Feng K., Shi X. (2021). Ultra-sensitive detection of ampicillin via dual-enzyme mediated cascade-signal amplified aptasensor. Microchem. J..

[27] Botto M.M., Murthy S., Lamers M.H. (2023). High-Throughput Exonuclease Assay Based on the Fluorescent Base Analogue 2-Aminopurine. ACS Omega.

[28] Russel N.S., Kodali G., Stanley R.J., Narayanan M. (2023). Screening for Novel Fluorescent Nucleobase Analogues Using Computational and Experimental Methods: 2-Amino-6-chloro-8-vinylpurine (2A6Cl8VP) as a Case Study. J. Phys. Chem. B.

[29] Kenfack C.A., Piémont E., Ben Gaied N., Burger A., Mély Y. (2008). Time-Resolved Fluorescent Properties of 8-Vinyl-deoxyadenosine and 2-Amino-deoxyribosylpurine Exhibit Different Sensitivity to Their Opposite Base in Duplexes. J. Phys. Chem. B.

[30] Caldero-Rodríguez N.E., Ortiz-Rodríguez L.A., Gonzalez A.A., Crespo-Hernández C.E. (2022). Photostability of 2,6-diaminopurine and its 2′-deoxyriboside investigated by femtosecond transient absorption spectroscopy. Phys. Chem. Chem. Phys..

[31] Albrecht C.S., Scatena L.F., von Hippel P.H., Marcus A.H. (2024). Two-photon excitation two-dimensional fluorescence spectroscopy (2PE-2DFS) of the fluorescent nucleobase 6-MI. Proc. SPIE.

[32] Dumas A., Luedtke N.W. (2011). Fluorescence Properties of 8-(2-Pyridyl) guanine “2PyG” as Compared to 2-Aminopurine in DNA. ChemBioChem.

[33] Passow K.T., Harki D.A. (2018). 4-Cyanoindole-2′-deoxyribonucleoside (4CIN): A Universal Fluorescent Nucleoside Analogue. Org. Lett..

[34] Yan Y., Tao H., He J., Huang S.-Y. (2020). The HDOCK server for integrated protein–protein docking. Nat. Protoc..

[35] Rigsby R.E., Parker A.B. (2016). Using the PyMOL application to reinforce visual understanding of protein structure. Biochem. Mol. Biol. Educ..

[36] Zhou W., Ding J., Liu J. (2017). 2-Aminopurine-modified DNA homopolymers for robust and sensitive detection of mercury and silver. Biosens. Bioelectron..

[37] Paterson K.A., Arlt J., Jones A.C. (2020). Dynamic and static quenching of 2-aminopurine fluorescence by the natural DNA nucleotides in solution. Methods Appl. Fluoresc..

[38] Jean J.M., Hall K.B. (2001). 2-Aminopurine fluorescence quenching and lifetimes: Role of base stacking. Proc. Natl. Acad. Sci. USA.

[39] Somsen O.J.G., Hoek V.A., Amerongen V.H. (2005). Fluorescence quenching of 2-aminopurine in dinucleotides. Chem. Phys. Lett..

[40] Wang X., Zeng R., Chu S., Tang W., Lin N., Fu J., Yang J., Gao B. (2019). A quencher-free DNAzyme beacon for fluorescently sensing uranyl ions via embedding 2-aminopurine. Biosens. Bioelectron..

[41] Hardman S.J.O., Thompson K.C. (2007). The fluorescence transition of 2-aminopurine in double- and single-stranded DNA. Int. J. Quantum Chem..

[42] Xiao X., Tao J., Zhang H.Z., Huang C.Z., Zhen S.J. (2016). Exonuclease III-assisted graphene oxide amplified fluorescence anisotropy strategy for ricin detection. Biosens. Bioelectron..

[43] Cui L., Ke G., Zhang W.Y., Yang C.J. (2011). A universal platform for sensitive and selective colorimetric DNA detection based on Exo III assisted signal amplification. Biosens. Bioelectron..

[44] Yoshida W., Mochizuki E., Takase M., Hasegawa H., Morita Y., Yamazaki H., Sode K., Ikebukuro K. (2009). Selection of DNA aptamers against insulin and construction of an aptameric enzyme subunit for insulin sensing. Biosens. Bioelectron..

[45] Dyakonova E.S., Koval V.V., Lomzov A.A., Ishchenko A.A., Fedorova O.S. (2018). Data on PAGE analysis and MD simulation for the interaction of endonuclease Apn1 from Saccharomyces cerevisiae with DNA substrates containing 5,6-dihydrouracyl and 2-aminopurine. Data Brief.

[46] Reha-Krantz L.J. (2009). The use of 2-aminopurine fluorescence to study DNA polymerase function. Methods Mol. Biol..

[47] Cho H., Kumar S., Yang D., Vaidyanathan S., Woo K., Garcia I., Shue H.J., Yoon Y., Ferreri K., Choo H. (2018). Surface-Enhanced Raman Spectroscopy-Based Label-Free Insulin Detection at Physiological Concentrations for Analysis of Islet Performance. ACS Sens..

[48] Taghdisi S.M., Danesh N.M., Lavaee P., Sarreshtehdar Emrani A., Ramezani M., Abnous K. (2015). Aptamer Biosensor for Selective and Rapid Determination of Insulin. Anal. Lett..

[49] He Y., Cheng Y., Wen X. (2022). A design of red emission CDs-based aptasensor for sensitive detection of insulin via fluorescence resonance energy transfer. Spectrochim. Acta Part A Mol. Biomol. Spectrosc..

[50] Park Y.M., Choi Y.S., Lee H.-R., Heo Y.S., Bae N.H., Lee T.J., Lee S.J. (2020). Flexible and highly ordered nanopillar electrochemical sensor for sensitive insulin evaluation. Biosens. Bioelectron..

[51] Ito E., Kaneda M., Kodama H., Morikawa M., Tai M., Aoki K., Miura T. (2015). Immunoreactive insulin in diabetes mellitus patient sera detected by ultrasensitive ELISA with thio-nad cycling. Biotechniques.

[52] Syed Z.U.Q., Samaraweera S., Wang Z., Krishnan S. (2024). Colorimetric nano-biosensor for low-resource settings: Insulin as a model biomarker. Sens. Diagn..

[53] Cappellani D., Macchia E., Falorni A., Marchetti P. (2020). Insulin Autoimmune Syndrome (Hirata Disease): A Comprehensive Review Fifty Years After Its First Description. Diabetes Metab. Syndr. Obes..

[54] Rungsima T., Rungpailin B., Sirirat P., Raweewan L., Apiradee S. (2017). Rare cause of recurrent hypoglycemia: Insulin autoimmune syndrome. Case Rep. Endocrinol..

[55] Stanton A.M., Stamatiades G.A. (2026). Insulin autoimmune syndrome presenting with recurrent hypoglycemia and extreme hyperinsulinemia. Ann. Intern. Med. Clin. Cases.

[56] Abraham L.A., Khalid S., Halik A., Malek R. (2024). Insulin autoimmune syndrome (hirata’s disease) caused by methimazole in a 47-year-old man. Cureus.

[57] Maheshvare M.D., Raha S., Knig M., Pal D. (2023). A pathway model of glucose-stimulated insulin secretion in the pancreatic β-cell. Front. Endocrinol..

[58] Zhang F., Tzanakakis E.S. (2017). Optogenetic regulation of insulin secretion in pancreatic β-cells. Sci. Rep..

[59] Gautheron G., Péraldi-Roux S., Vaillé J., Belhadj S., Patyra A., Bayle M., Youl E., Omhmmed S., Guyot M., Cros G. (2025). The flavonoid resokaempferol improves insulin secretion from healthy and dysfunctional pancreatic β-cells. Br. J. Pharmacol..

