# Supplementary material for: An Ultrasensitive Label-Free Aptasensor for Insulin Detection Assisted by Exonuclease III and 2-Aminopurine
Source: Molecules. 2026 Jun 21;31(12):2173. doi: 10.3390/molecules31122173 (PMC13304847; doi:10.3390/molecules31122173)
Supplement: Supplementary file 1 [file molecules-31-02173-s001.zip › molecules-4345651-Supplementary.pdf]

An ultrasensitive label-free aptasensor for insulin detection assisted by exonuclease III and 2-aminopurine

Dongdong Shi[a], Yanhua He[b], Guiqin Yan[c, *]

[a] School of Chemistry and Chemical Engineering, Shanxi Normal University,Taiyuan 030031, PR China，118111005@stu.sxnu.edu.cn

[b]School of Food Science, Shanxi Normal University, Taiyuan 030031, PR China

c[*]School of Life Sciences, Shanxi Normal University,Taiyuan 030031, PR China，gqinyan2013@126.com

S1. Insulin detection

Probe hybridization: 17.5 μL of 10 μM 2-aminopurine (2AP)-labeled hairpin probe and 17.5 μL of 10 μM complementary DNA (cDNA) were sequentially transferred into a 10 mL colorimetric tube. The mixture was blended with a small portion of Tris-HCl buffer and incubated at 37°C for 20 min. Hybridization yielded blunt-ended hairpin duplexes, which effectively quenched the intrinsic fluorescence of 2AP.

Insulin incubation: Gradient volumes of 10 μM insulin stock solution were introduced into the above mixtures to achieve final insulin concentrations of 0, 3, 6, 10, 25, 35, 50, 70, 90, 110, 120 and130 nM. The corresponding injection volumes were 0, 1.5, 3.0, 5.0, 12.5, 17.5, 25, 35, 45, 55, 60 and 65μL, respectively. After thorough homogenization, the solutions were incubated at room temperature for 20 min. The specific binding between insulin and the aptamer sealed the termini of nucleic acids and thus protected the DNA from Exo Ⅲ-mediated cleavage.

Enzymatic digestion: Subsequently, 25 μL of 1M $MgCl_2$ aqueous solution and 5 μL of Exo Ⅲ stock solution (200U/μL) were supplemented. The resultant mixture was incubated in a thermostatted water bath at 37 °C for 120 min. The hairpin DNA unprotected by insulin was degraded by Exo Ⅲ, accompanied by fluorescence recovery of the released 2AP.

Volume adjustment and fluorescence measurement: All reaction systems were brought to a final volume of 5 mL with pH 7.4 Tris-HCl buffer. Fluorescence signals were detected in triplicate for each sample.

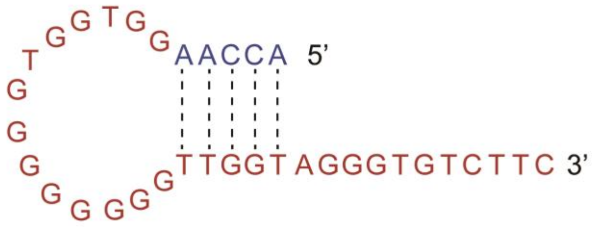

Figure. S1 Secondary structure of 2AP-hairpin probe

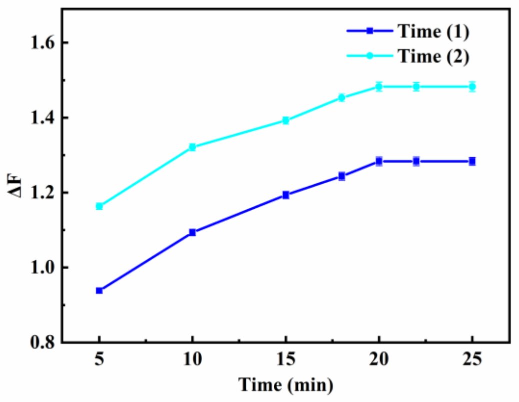

Figure. S2 The best reaction times for the 2AP-hairpin probe to cDNA coupling (time 1) and aptamer binding of Ins (time 2)

Table S1 Comparison with commercial colorimetric enzyme-linked immunosorbent assay (ELISA) kits for insulin detection

Approach	Limit of Detection (LOD)	Linear Range	Assay Time	Ref. Company
Mercodia rat insulin ELISA (HRP-label)	20 nM	20-100 nM	2 .5h	Mercodia
Ultrasensitive thio-NAD cycling ELISA (ALP-label)	2.28 pM	0–48 pM	12 h	1
Human insulin ELISA kit (NBP2-60077, HRP-label)	12 pM	60–359 pM	2 h	Novus Biologicals
Insulin ELISA (80-INSHU-E01.1, E10.1)	2.4 pM	18–1198 pM	2 h	ALPCO
Human insulin ELISA kit (ab100578, biotin-streptavidin-HRP label)	24 pM	28–1798 pM	5 h	Abcam
Colorimetric nano-biosensor (sandwich-HRP-label based)	10 pM	10 pM–10 nM	2 h	2
Fluorescence	1.62 nM	3–130nM	2.67h	This work

References

51. Ito, E.; Kaneda, M.; Kodama, H.; Morikawa, M.; Tai, M.; Aoki, K.; Miura, T. Immunoreactive insulin in diabetes mellitus patient sera detected by ultrasensitive ELISA with thio-nad cycling. *Biotechniques* **2015**, *59*, 359–361. https://doi.org/10.2144/000114355.

52. Syed, Z.U.Q.; Samaraweera, S.; Wang, Z.; Krishnan, S. Colorimetric nano-biosensor for low-resource settings: Insulin as a model biomarker. *Sens. Diagn.* **2024**, *3*, 1659–1671. https://doi.org/10.1039/d4sd00197d.
